# Bis(2-{[(9*H*-fluoren-2-yl)methyl­idene]amino}­phenolato-κ^2^ *N*,*O*)zinc methanol disolvate

**DOI:** 10.1107/S160053681201272X

**Published:** 2012-03-31

**Authors:** Young-Inn Kim, Sung-Jae Yun, Inn-Hye Hwang, Dae-Young Kim, Sung Kwon Kang

**Affiliations:** a Department of Chemistry Education and Interdisciplinary Program of Advanced Information and Display Materials, Pusan National University, Busan 609-735, Republic of Korea; b Department of Chemistry, Chungnam National University, Daejeon 305-764, Republic of Korea

## Abstract

In the title compound, [Zn(C_20_H_14_NO)_2_]·2CH_3_OH, the Zn^II^ atom lies on a crystallographic twofold rotation axis and is coordinated by two O atoms and two N atoms from two bidentate 2-{[(9*H*-fluoren-2-yl)methyl­idene]amino}­phenolate ligands within a distorted tetra­hedral geometry. The dihedral angle between the two chelate rings is 82.92 (5)°. In the coordinated ligand, the phenol ring is twisted at 30.22 (9)° from the mean plane of the fluorene ring. In the crystal, O—H⋯O hydrogen bonds link the complex mol­ecules to the methanol solvent mol­ecules.

## Related literature

For general background to Schiff base complexes, see: Ji *et al.* (2012 ▶); Niu *et al.* (2012 ▶); Liu *et al.* (2011 ▶); Roy *et al.* (2009 ▶). For the structures and luminescent properties of Hg(II) complexes, see: Kim *et al.* (2011 ▶); Kim & Kang (2010 ▶). For the physical properties of fluorene complexes, see: Scaria *et al.* (2010 ▶); Loy *et al.* (2002 ▶); Miteva *et al.* (2001 ▶).
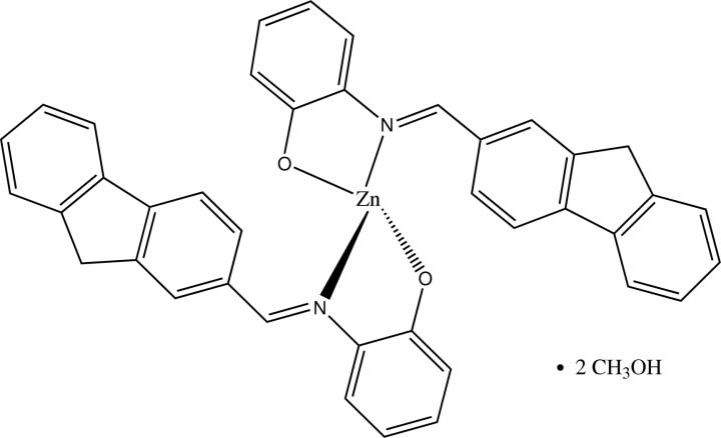


## Experimental

### 

#### Crystal data

[Zn(C_20_H_14_NO)_2_]·2CH_4_O
*M* *_r_* = 698.1
Monoclinic, 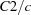

*a* = 13.7294 (3) Å
*b* = 13.9123 (2) Å
*c* = 18.8383 (3) Å
β = 110.652 (1)°
*V* = 3367.03 (10) Å^3^
*Z* = 4
Mo *K*α radiation
μ = 0.78 mm^−1^
*T* = 296 K
0.10 × 0.05 × 0.04 mm

#### Data collection

Bruker SMART CCD area-detector diffractometer
Absorption correction: multi-scan (*SADABS*; Bruker, 2002 ▶) *T*_min_ = 0.922, *T*_max_ = 0.966
10682 measured reflections
3078 independent reflections
2134 reflections with *I* > 2σ(*I*)
*R*_int_ = 0.039

#### Refinement

*R*[*F* ^2^ > 2σ(*F* ^2^)] = 0.043
*wR*(*F* ^2^) = 0.096
*S* = 1.02
3078 reflections
227 parameters
H atoms treated by a mixture of independent and constrained refinement
Δρ_max_ = 0.22 e Å^−3^
Δρ_min_ = −0.32 e Å^−3^

### 

Data collection: *SMART* (Bruker, 2002 ▶); cell refinement: *SAINT* (Bruker, 2002 ▶); data reduction: *SAINT*; program(s) used to solve structure: *SHELXS97* (Sheldrick, 2008 ▶); program(s) used to refine structure: *SHELXL97* (Sheldrick, 2008 ▶); molecular graphics: *ORTEP-3 for Windows* (Farrugia, 1997 ▶); software used to prepare material for publication: *WinGX* (Farrugia, 1999 ▶).

## Supplementary Material

Crystal structure: contains datablock(s) global, I. DOI: 10.1107/S160053681201272X/tk5073sup1.cif

Structure factors: contains datablock(s) I. DOI: 10.1107/S160053681201272X/tk5073Isup2.hkl

Additional supplementary materials:  crystallographic information; 3D view; checkCIF report

## Figures and Tables

**Table:** 

Zn1—O1	1.9239 (17)
Zn1—N8	2.052 (2)
C7—N8	1.433 (3)
N8—C9	1.293 (3)

**Table:** 

O1^i^—Zn1—O1	115.61 (11)
O1^i^—Zn1—N8	125.99 (8)
O1—Zn1—N8	85.68 (8)
N8—Zn1—N8^i^	122.53 (11)

Symmetry code: (i) 
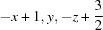
.

**Table 2:** Hydrogen-bond geometry (Å, °)

*D*—H⋯*A*	*D*—H	H⋯*A*	*D*⋯*A*	*D*—H⋯*A*
O24—H24⋯O1	0.98 (4)	1.82 (5)	2.794 (3)	173 (4)

**Fig. 1.**
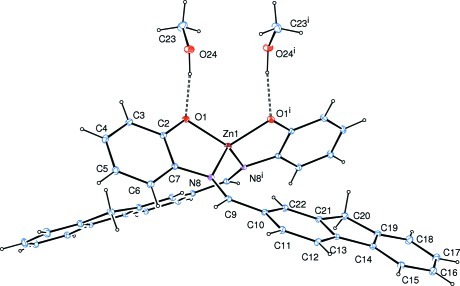
Molecular structure of the title compound, showing the atom-numbering scheme and 30% probability ellipsoids. The O—H···O hydrogen bonds are indicated by dashed lines. [symmetry code: (i) -x + 1, y, -z + 3/2].

## supplementary crystallographic information

### Comment

Schiff base ligands have attracted attention due to their facile syntheses, easily tunable steric and electronic properties resulting in a good performance in sensor technologies and electroluminescence devices (Ji *et al.*, 2012; Niu *et al.*, 2012; Liu *et al.*, 2011; Roy *et al.*, 2009). Recently, we reported group 12, Hg(II) complexes with Schiff bases with an emphasis on their luminescent properties (Kim & Kang, 2010; Kim *et al.*, 2011). Herein, we designed new Schiff base containing a fluorene moiety since flourene has high triplet energy and good-hole transporting ability (Scaria *et al.*, 2010; Loy *et al.*, 2002; Miteva *et al.*, 2001) and synthesized its Zn(II) complex. The title compound shows a red emission at 611 nm with a quantum yield of 1.2% in a DMF solution upon 300 nm excitation.

In (I), Fig. 1, the Zn^II^ atom lies on a twofold axis and is coordinated by two O atoms and two N atoms of two bidentate 2-((9*H*-fluoren-2-yl)methyleneamino)phenolato ligands in a distorted tetrahedral geometry. The angles around Zn atom are within the range of 85.68 (8)–125.99 (8) ° (Table 1). The dihedral angle between the O1/Zn1/N8 and O1^i^/Zn1/N1^i^ [symmetry code: (i) -*x* + 1, *y*, -*z* + 3/2] is 83.48 (6) °. The fluorene moiety (C9—C22) is almost planar, with r.m.s. deviations of 0.018 Å from the corresponding least-squares plane defined by the thirteen constituent atoms. In the 2-((9*H*-fluoren-2-yl)methyleneamino)phenol ligand, the phenol ring (O1–C7) is twisted at 30.22 (9) ° from the mean plane of the fluorene ring. The presence of intermolecular O24—H24···O1 hydrogen bonds link the complex and solvent molecules (Table 2 and Fig. 1).

### Experimental

Preparation of ligand: 9*H*-fluorene-2-carbaldehyde (1.94 g, 10 mmol) was slowly added to 2-aminophenol (1.09 g, 10 mmol) in methanol/methylenechloride (1:1 *v*/*v*) (40 ml) solution at 50 °C for 5 h to obtain (*E*)-2-((9*H*-fluoren-2-yl)methyleneamino)phenol (*L*) as a yellow powder. Yield: 1.55 g (75%).

Preparation of zinc(II) compound: the reaction of *L* compound (1.50 g, 10 mmol) with zinc(II) acetate (0.91 g, 5 mmol) in methanol/methylenechloride (1:1 *v*/*v*) (40 ml) at 50 °C for 5 h yielded the title compound as an orange powder. The powder was filtered off and washed with hexane. Yield: 0.95 g (30%). Orange crystals of (I) were obtained from its ethanol solution by slow evaporation of the solvent at room temperature.

### Refinement

Atom H24 of the OH group was located from a difference Fourier map and refined freely [refined distance; O—H = 0.98 (4) Å]. Other H atoms were positioned geometrically and refined using a riding model, with C—H = 0.93 – 0.97 Å with *U*_iso_(H) = 1.2*U*_eq_(carrier C) for aromatic- and methylene-H, and 1.5*U*_eq_(carrier C) for methyl-H atoms.

### Crystal data

**Table:**

[Zn(C_20_H_14_NO)_2_]·2CH_4_O	*F*(000) = 1456
*M**_r_* = 698.1	*D*_x_ = 1.377 Mg m^−^^3^
Monoclinic, *C*2/*c*	Mo *K*α radiation, λ = 0.71073 Å
Hall symbol: -C 2yc	Cell parameters from 2869 reflections
*a* = 13.7294 (3) Å	θ = 2.2–25.3°
*b* = 13.9123 (2) Å	µ = 0.78 mm^−^^1^
*c* = 18.8383 (3) Å	*T* = 296 K
β = 110.652 (1)°	Block, orange
*V* = 3367.03 (10) Å^3^	0.1 × 0.05 × 0.04 mm
*Z* = 4	

### Data collection

**Table:**

Bruker SMART CCD area-detector diffractometer	2134 reflections with *I* > 2σ(*I*)
Graphite monochromator	*R*_int_ = 0.039
φ and ω scans	θ_max_ = 25.5°, θ_min_ = 2.2°
Absorption correction: multi-scan (*SADABS*; Bruker, 2002)	*h* = −9→16
*T*_min_ = 0.922, *T*_max_ = 0.966	*k* = −13→16
10682 measured reflections	*l* = −22→16
3078 independent reflections	

### Refinement

**Table:**

Refinement on *F*^2^	Primary atom site location: structure-invariant direct methods
Least-squares matrix: full	Secondary atom site location: difference Fourier map
*R*[*F*^2^ > 2σ(*F*^2^)] = 0.043	Hydrogen site location: inferred from neighbouring sites
*wR*(*F*^2^) = 0.096	H atoms treated by a mixture of independent and constrained refinement
*S* = 1.02	*w* = 1/[σ^2^(*F*_o_^2^) + (0.0431*P*)^2^ + 0.3461*P*] where *P* = (*F*_o_^2^ + 2*F*_c_^2^)/3
3078 reflections	(Δ/σ)_max_ < 0.001
227 parameters	Δρ_max_ = 0.22 e Å^−^^3^
0 restraints	Δρ_min_ = −0.32 e Å^−^^3^

### Special details

**Table:**

Geometry. All s.u.'s (except the s.u. in the dihedral angle between two l.s. planes) are estimated using the full covariance matrix. The cell s.u.'s are taken into account individually in the estimation of s.u.'s in distances, angles and torsion angles; correlations between s.u.'s in cell parameters are only used when they are defined by crystal symmetry. An approximate (isotropic) treatment of cell s.u.'s is used for estimating s.u.'s involving l.s. planes.
Refinement. Refinement of *F*^2^ against ALL reflections. The weighted *R*-factor *wR* and goodness of fit *S* are based on *F*^2^, conventional *R*-factors *R* are based on *F*, with *F* set to zero for negative *F*^2^. The threshold expression of *F*^2^ > 2σ(*F*^2^) is used only for calculating *R*-factors(gt) *etc*. and is not relevant to the choice of reflections for refinement. *R*-factors based on *F*^2^ are statistically about twice as large as those based on *F*, and *R*- factors based on ALL data will be even larger.

### Fractional atomic coordinates and isotropic or equivalent isotropic displacement parameters (Å^2^)

**Table:**

	*x*	*y*	*z*	*U*_iso_*/*U*_eq_	
Zn1	0.5	0.57842 (3)	0.75	0.04130 (18)	
O1	0.40411 (14)	0.65210 (12)	0.66885 (10)	0.0454 (5)	
C2	0.3082 (2)	0.61436 (18)	0.64660 (16)	0.0400 (7)	
C3	0.2277 (2)	0.6498 (2)	0.58475 (17)	0.0515 (8)	
H3	0.2406	0.7007	0.5573	0.062*	
C4	0.1294 (3)	0.6119 (2)	0.56296 (17)	0.0558 (8)	
H4	0.0765	0.6381	0.5217	0.067*	
C5	0.1082 (2)	0.5351 (2)	0.60185 (17)	0.0558 (8)	
H5	0.0419	0.5083	0.586	0.067*	
C6	0.1861 (2)	0.4984 (2)	0.66416 (16)	0.0499 (8)	
H6	0.1721	0.4467	0.6903	0.06*	
C7	0.2851 (2)	0.53798 (18)	0.68829 (15)	0.0380 (7)	
N8	0.37074 (17)	0.50751 (14)	0.75354 (12)	0.0372 (5)	
C9	0.3537 (2)	0.45901 (18)	0.80656 (16)	0.0433 (7)	
H9	0.2846	0.4457	0.7997	0.052*	
C10	0.4315 (2)	0.42353 (17)	0.87534 (15)	0.0410 (7)	
C11	0.3957 (2)	0.38990 (19)	0.93208 (16)	0.0468 (7)	
H11	0.3245	0.3882	0.9225	0.056*	
C12	0.4632 (2)	0.35952 (18)	1.00140 (16)	0.0456 (7)	
H12	0.4382	0.3384	1.0386	0.055*	
C13	0.5689 (2)	0.36100 (17)	1.01473 (15)	0.0404 (7)	
C14	0.6578 (2)	0.33320 (18)	1.08148 (15)	0.0414 (7)	
C15	0.6623 (3)	0.29326 (19)	1.15031 (17)	0.0507 (8)	
H15	0.6014	0.2809	1.1597	0.061*	
C16	0.7570 (3)	0.2723 (2)	1.20397 (18)	0.0619 (9)	
H16	0.7606	0.2456	1.2501	0.074*	
C17	0.8467 (3)	0.2908 (2)	1.1897 (2)	0.0730 (10)	
H17	0.9106	0.2766	1.2269	0.088*	
C18	0.8446 (3)	0.3300 (2)	1.1216 (2)	0.0697 (10)	
H18	0.906	0.342	1.1129	0.084*	
C19	0.7496 (2)	0.35077 (19)	1.06721 (17)	0.0510 (8)	
C20	0.7238 (2)	0.3916 (2)	0.98824 (17)	0.0548 (8)	
H20A	0.7525	0.4556	0.9899	0.066*	
H20B	0.75	0.3506	0.9574	0.066*	
C21	0.6071 (2)	0.39376 (18)	0.95847 (16)	0.0411 (7)	
C22	0.5384 (2)	0.42345 (18)	0.88995 (16)	0.0456 (7)	
H22	0.5633	0.4439	0.8525	0.055*	
C23	0.4138 (3)	0.9024 (3)	0.6538 (2)	0.0906 (12)	
H23A	0.3826	0.8908	0.6003	0.136*	
H23B	0.4867	0.8876	0.6704	0.136*	
H23C	0.4049	0.9688	0.6641	0.136*	
O24	0.36624 (18)	0.84464 (18)	0.69240 (13)	0.0716 (7)	
H24	0.385 (3)	0.778 (3)	0.687 (2)	0.141 (17)*	

### Atomic displacement parameters (Å^2^)

**Table:**

	*U*^11^	*U*^22^	*U*^33^	*U*^12^	*U*^13^	*U*^23^
Zn1	0.0383 (3)	0.0397 (3)	0.0464 (3)	0	0.0155 (2)	0
O1	0.0421 (13)	0.0433 (11)	0.0542 (12)	−0.0024 (9)	0.0210 (10)	0.0088 (9)
C2	0.0394 (19)	0.0400 (15)	0.0429 (18)	0.0036 (14)	0.0176 (16)	0.0002 (14)
C3	0.052 (2)	0.0487 (17)	0.053 (2)	0.0051 (16)	0.0180 (18)	0.0143 (15)
C4	0.046 (2)	0.064 (2)	0.0497 (19)	0.0113 (17)	0.0070 (17)	0.0078 (16)
C5	0.044 (2)	0.066 (2)	0.054 (2)	−0.0049 (17)	0.0124 (18)	0.0040 (17)
C6	0.046 (2)	0.0517 (17)	0.0512 (19)	−0.0036 (16)	0.0163 (17)	0.0059 (15)
C7	0.0383 (18)	0.0370 (14)	0.0400 (16)	0.0024 (13)	0.0155 (15)	−0.0011 (13)
N8	0.0392 (14)	0.0354 (12)	0.0392 (13)	0.0002 (11)	0.0165 (12)	0.0007 (11)
C9	0.0421 (19)	0.0404 (15)	0.0502 (19)	−0.0046 (14)	0.0197 (16)	−0.0011 (14)
C10	0.0484 (19)	0.0363 (14)	0.0405 (17)	−0.0011 (14)	0.0184 (15)	0.0020 (14)
C11	0.0410 (19)	0.0538 (17)	0.0475 (19)	−0.0024 (14)	0.0178 (16)	0.0054 (15)
C12	0.047 (2)	0.0503 (17)	0.0452 (18)	0.0003 (15)	0.0232 (16)	0.0069 (14)
C13	0.045 (2)	0.0310 (14)	0.0466 (18)	−0.0013 (14)	0.0177 (16)	−0.0020 (13)
C14	0.046 (2)	0.0336 (15)	0.0435 (17)	0.0020 (13)	0.0141 (16)	0.0006 (13)
C15	0.054 (2)	0.0461 (17)	0.051 (2)	0.0051 (15)	0.0167 (18)	0.0000 (15)
C16	0.070 (3)	0.058 (2)	0.053 (2)	0.0075 (19)	0.015 (2)	0.0081 (16)
C17	0.052 (2)	0.078 (2)	0.070 (2)	0.0054 (19)	−0.002 (2)	0.016 (2)
C18	0.047 (2)	0.078 (2)	0.076 (3)	−0.0037 (18)	0.013 (2)	0.017 (2)
C19	0.046 (2)	0.0468 (17)	0.057 (2)	−0.0035 (15)	0.0131 (18)	0.0079 (15)
C20	0.045 (2)	0.0551 (18)	0.067 (2)	−0.0028 (15)	0.0223 (17)	0.0119 (16)
C21	0.0400 (18)	0.0382 (15)	0.0450 (18)	−0.0013 (13)	0.0151 (16)	0.0045 (13)
C22	0.050 (2)	0.0434 (16)	0.0505 (19)	−0.0010 (15)	0.0262 (17)	0.0063 (15)
C23	0.076 (3)	0.095 (3)	0.102 (3)	−0.004 (2)	0.032 (3)	0.034 (2)
O24	0.0702 (17)	0.0578 (14)	0.0988 (18)	−0.0035 (12)	0.0448 (15)	0.0010 (13)

### Geometric parameters (Å, º)

**Table:**

Zn1—O1^i^	1.9239 (18)	C12—H12	0.93
Zn1—O1	1.9239 (17)	C13—C21	1.413 (3)
Zn1—N8	2.052 (2)	C13—C14	1.462 (4)
Zn1—N8^i^	2.052 (2)	C14—C15	1.392 (4)
O1—C2	1.341 (3)	C14—C19	1.399 (4)
C2—C3	1.383 (4)	C15—C16	1.367 (4)
C2—C7	1.422 (4)	C15—H15	0.93
C3—C4	1.370 (4)	C16—C17	1.374 (4)
C3—H3	0.93	C16—H16	0.93
C4—C5	1.384 (4)	C17—C18	1.385 (4)
C4—H4	0.93	C17—H17	0.93
C5—C6	1.376 (4)	C18—C19	1.376 (4)
C5—H5	0.93	C18—H18	0.93
C6—C7	1.387 (4)	C19—C20	1.513 (4)
C6—H6	0.93	C20—C21	1.499 (4)
C7—N8	1.433 (3)	C20—H20A	0.97
N8—C9	1.293 (3)	C20—H20B	0.97
C9—C10	1.445 (4)	C21—C22	1.367 (4)
C9—H9	0.93	C22—H22	0.93
C10—C22	1.395 (4)	C23—O24	1.392 (4)
C10—C11	1.404 (3)	C23—H23A	0.96
C11—C12	1.374 (4)	C23—H23B	0.96
C11—H11	0.93	C23—H23C	0.96
C12—C13	1.384 (4)	O24—H24	0.98 (4)
			
O1^i^—Zn1—O1	115.61 (11)	C12—C13—C21	120.8 (3)
O1^i^—Zn1—N8	125.99 (8)	C12—C13—C14	131.0 (3)
O1—Zn1—N8	85.68 (8)	C21—C13—C14	108.2 (3)
O1^i^—Zn1—N8^i^	85.68 (8)	C15—C14—C19	120.1 (3)
O1—Zn1—N8^i^	125.99 (8)	C15—C14—C13	131.0 (3)
N8—Zn1—N8^i^	122.53 (11)	C19—C14—C13	108.9 (2)
C2—O1—Zn1	111.18 (15)	C16—C15—C14	119.5 (3)
O1—C2—C3	121.9 (3)	C16—C15—H15	120.3
O1—C2—C7	120.4 (3)	C14—C15—H15	120.3
C3—C2—C7	117.7 (3)	C15—C16—C17	120.0 (3)
C4—C3—C2	121.7 (3)	C15—C16—H16	120
C4—C3—H3	119.2	C17—C16—H16	120
C2—C3—H3	119.2	C16—C17—C18	121.8 (3)
C3—C4—C5	120.5 (3)	C16—C17—H17	119.1
C3—C4—H4	119.8	C18—C17—H17	119.1
C5—C4—H4	119.8	C19—C18—C17	118.5 (3)
C6—C5—C4	119.5 (3)	C19—C18—H18	120.7
C6—C5—H5	120.2	C17—C18—H18	120.7
C4—C5—H5	120.2	C18—C19—C14	120.1 (3)
C5—C6—C7	120.6 (3)	C18—C19—C20	130.0 (3)
C5—C6—H6	119.7	C14—C19—C20	109.9 (3)
C7—C6—H6	119.7	C21—C20—C19	102.9 (2)
C6—C7—C2	119.9 (3)	C21—C20—H20A	111.2
C6—C7—N8	125.2 (2)	C19—C20—H20A	111.2
C2—C7—N8	114.8 (2)	C21—C20—H20B	111.2
C9—N8—C7	120.0 (2)	C19—C20—H20B	111.2
C9—N8—Zn1	132.1 (2)	H20A—C20—H20B	109.1
C7—N8—Zn1	106.68 (16)	C22—C21—C13	119.4 (3)
N8—C9—C10	126.4 (3)	C22—C21—C20	130.6 (3)
N8—C9—H9	116.8	C13—C21—C20	110.0 (3)
C10—C9—H9	116.8	C21—C22—C10	121.0 (2)
C22—C10—C11	118.4 (3)	C21—C22—H22	119.5
C22—C10—C9	124.8 (2)	C10—C22—H22	119.5
C11—C10—C9	116.7 (3)	O24—C23—H23A	109.5
C12—C11—C10	121.7 (3)	O24—C23—H23B	109.5
C12—C11—H11	119.1	H23A—C23—H23B	109.5
C10—C11—H11	119.1	O24—C23—H23C	109.5
C11—C12—C13	118.7 (3)	H23A—C23—H23C	109.5
C11—C12—H12	120.6	H23B—C23—H23C	109.5
C13—C12—H12	120.6	C23—O24—H24	108 (2)

Symmetry code: (i) −*x*+1, *y*, −*z*+3/2.

### Hydrogen-bond geometry (Å, º)

**Table:**

*D*—H···*A*	*D*—H	H···*A*	*D*···*A*	*D*—H···*A*
O24—H24···O1	0.98 (4)	1.82 (5)	2.794 (3)	173 (4)
